# Fracture reduction with positive medial cortical support: a key element in stability reconstruction for the unstable pertrochanteric hip fractures

**DOI:** 10.1007/s00402-015-2206-x

**Published:** 2015-04-04

**Authors:** Shi-Min Chang, Ying-Qi Zhang, Zhuo Ma, Qing Li, Jens Dargel, Peer Eysel

**Affiliations:** 1 The Department of Orthopaedic Surgery, Yangpu Hospital, Tongji University School of Medicine, 450 Tengyue Road, Shanghai, 200090 People’s Republic of China; 2 Department of Orthopaedic and Trauma Surgery, Cologne University Hospital, Cologne, Germany

**Keywords:** Pertrochanteric fracture, Fracture reduction, Positive medial cortical support, Cephalomedullary nail, Wedge-open effect, Hip–thigh pain, Secondary stability

## Abstract

**Purpose:**

To introduce the concept of fracture reduction with positive medial cortical support and its clinical and radiological correlation in geriatric unstable pertrochanteric fractures.

**Methods:**

A retrospective analysis of 127 patients (32 men and 95 women, with mean age 78.7 years) with AO/OTA 31A2.2 and 2.3 hip fractures treated with cephalomedullary nail (PFNA-II or Gamma-3) between July 2010 and June 2013 was performed. They were classified into three groups according the grade of medial cortical support in postoperative fracture reduction (positive, neutral, and negative). The positive cortex support was defined that the medial cortex of the head–neck fragment displaced and located a little bit superomedially to the medial cortex of the shaft. If the neck cortex is located laterally to the shaft, it is negative with no cortical buttress, and if the two cortices contact smoothly, it is in neutral position. The demographic baseline, postoperative radiographic femoral neck–shaft angle and neck length, rehabilitation progress and functional recovery scores of each group were recorded and compared.

**Results:**

There were 89 cases (70 %) in positive, 26 in neutral, and 12 in negative support. No statistical differences were found between the three groups among patient age, sex ratio, prefracture score of activity of daily living, walking ability score, ASA physical risk score, number of medical comorbidities, osteoporosis Singh index, fracture reduction quality (Garden alignments), and the position of lag screw or helical blade in femoral head (TAD). In follow-up, patients in positive medial cortical support reduction group had the least loss in neck–shaft angle and neck length, and got ground-walking much earlier than negative reduction group, with good functional outcomes and less hip–thigh pain presence.

**Conclusion:**

Fracture reduction with nonanatomic positive medial cortical support allows limited sliding of the head–neck fragment to contact with the femur shaft and achieve secondary stability, providing a good mechanical environment for fracture healing.

## Introduction

Geriatric pertrochanteric hip fractures are still a major orthopedic challenge worldwide [1]. Despite the fact that fracture union rates are high, the functional outcomes tend to be disappointing [2–4]. A combination of factors, such as medical comorbidities, patient compliance, fracture pattern, quality of the bone, and environmental factors are thought to be responsible for this poor result [5–9]. Many of these factors cannot be addressed at the time of fracture presentation.

As the operative procedure is a major component in the treatment of patients with hip fractures, understanding the causes of failure is integral to any attempt to achieve an improved functional outcome. In 1980, Kaufer [10] described five major factors related to the treatment outcome, i.e. the bone quality, the fragment geometry, the choice of implant, the quality of reduction, and the placement of the implant in femoral head. However, the stability of the fracture after implant fixation is primarily dependent on the quality of fracture reduction. It is well known that slight valgus position to allow impaction means more stable fracture reduction and implies better outcome. Besides the valgus alignment, it is paramount important to achieve an anatomical contact between the anteromedial cortices of the two major fragments, the head–neck and the shaft [11–14].

In this paper, we describe the concept of positive medial cortical support (PMCS) in fracture reduction of unstable pertrochanteric fractures treated with cephalomedullary nail. PMCS is defined as the medial cortex of the head–neck fragment is displaced and located a little bit superomedially to the medial cortex of the femur shaft in AP view. PMCS reduction is a key element for stability reconstruction for unstable fractures, as it allows limited sliding of the head–neck fragment after operation (fracture impaction) to contact with the femur shaft and achieve secondary stability, providing a good mechanical environment for fracture healing. PMCS differs from the anatomic reduction of the anteromedial cortex. PMCS is a functional nonanatomic buttress reduction, which is easy to achieve in practice and is used for description of secondary stability after sliding impaction. While exact anatomic reduction is difficult to obtain and is used for primary fracture stability.

## Patients and methods

### Patient data collection

After Institutional Review Board approval, a retrospective analysis of 127 consecutive patients (32 men and 95 women) sustained pertrochanteric fractures from July 2010 to June 2013 was performed. One hundred and eleven patients were treated with PFNA-II, thirty-two with Gamma-3 nail. All the patients met the criteria as followed: (1) age 60 or older, (2) home accommodation before injury, (3) hip fractures of nonpathologic origin, (4) ambulatory without assistive devices before fracture, (5) no mental complications, (6) fracture type (AO/OTA classification 31A2.2 and 2.3 [15]), (6) follow-up for at least 6 months after operation.

Preinjury, surgery/anesthesia, postoperative course and follow-up data were collected for each patient [16]. (1) Preinjury data included age, gender, general physical condition (ASA grade), comorbidity (number and type: diabetes mellitus, hypertension, cancer, chronic obstructive pulmonary disease, cardiac arrhythmia, congestive heart failure, ischemic heart disease, cerebrovascular accident, renal disease, and disease need anticoagulant therapy), nutritional status (hemoglobin ≥90 g/l, albumin ≥35 g/l), the basic activity of daily life (BADL), the Parker mobility score [17] and osteoporosis (Singh index). (2) Surgical and anesthetic data included fracture type, duration of the operation, operative blood loss, blood transfusions, duration of anesthesia, and type of anesthesia. (3) Postoperative data included medical and surgical complications (lung infection, urinary infection, delirium, myocardial infarction, acute renal failure, acute heart failure, cerebrovascular accident, deep vein thrombosis, gastric stress ulcer and decubitus). Follow-up data included the timing of full-weight bearing walk, patient self-assessment, clinical and radiographic check at 3 and 6 months after surgery. Full-weight bearing was assessed by simply observing the patient walk, without any assistive device, or only one-hand stick was used for body balance.

### Surgical technique and perioperative management

The average time interval between injury and operation was 2.2 days (2–5 days). All procedures were performed with patients in the supine position on a fracture table under general or spinal anesthesia. Routine closed reduction maneuvers including abduction, traction and internal rotation was performed to get fracture alignment and confirmed by fluoroscopy (slight valgus in AP and <20° in lat). If closed reduction was not acceptable, especially in lateral sagittal view (for example, posterior sag or posterior neck displacement), intraoperative manipulation was performed later through the entry incision.

A nail entry site was created on the medial edge of the tip of the greater trochanter. The proximal part (no more than 2 cm) of the medullary canal was reamed. Short nails were used for all patients. As a general rule, if the patient body height was less than 160 cm, extra-small nail (170 mm length with 9 mm diameter) was chosen, if the body height was greater than 180 cm, normal nail (240 mm length with 10 mm diameter) was chosen, and if the body height was between 160 and 180 cm, small nail (200 mm length with 10 mm diameter) was selected. After the nail was inserted, it can be used as a tool to separate the engaged head–neck fragment from the shaft. By lateral pull of the nail jig, the fragments were loosened and sagittal reduction was easily manipulated by leverage technique using a bone hook or a long forceps [18].

For PFNA-II, the helical blade was attempted to be placed in the central of the femoral head both on anteroposterior (AP) and lateral view, while for the Gamma nail, the lag screw was in the lower third of the head on AP view and central on lateral view. Distal locking was performed with one screw in static mode.

No drainage was used after surgery. Blood transfusion was performed if the hemoglobin was below 90 g/l. Cefuroxime was used for 48 h postoperatively for prophylactic anti-infective therapy. Low molecular weight heparin was used for anticoagulation. No opioid analgesic was provided to the patients for the sake of its potential risks of cognitive impairment and respiratory depression.

Patients began isometric quadriceps exercises on the first day after surgery. About 10 days after surgery, partial weight bearing (just standing in bedside with bilateral feet) was allowed on the injured limb as tolerated by the patient. Physical therapists were also got involved to draw integral rehabilitation protocol. In follow-up, clinical and functional outcomes were assessed using the BADL and the Parker-Palmer mobility score.

### Radiological measurement

Standard AP radiographs of the hip were obtained with both legs positioned to an internal rotation of 15°. The lateral radiographs were taken with the contralateral hip flexed and abducted. The reduction quality was primarily categorized as good, acceptable, or poor using the method proposed by Baumgaertner et al. [19], including fragment alignment and displacement. The tip–apex distance (TAD) was measured from the immediate postoperative radiographs.

**Fig. 1:**
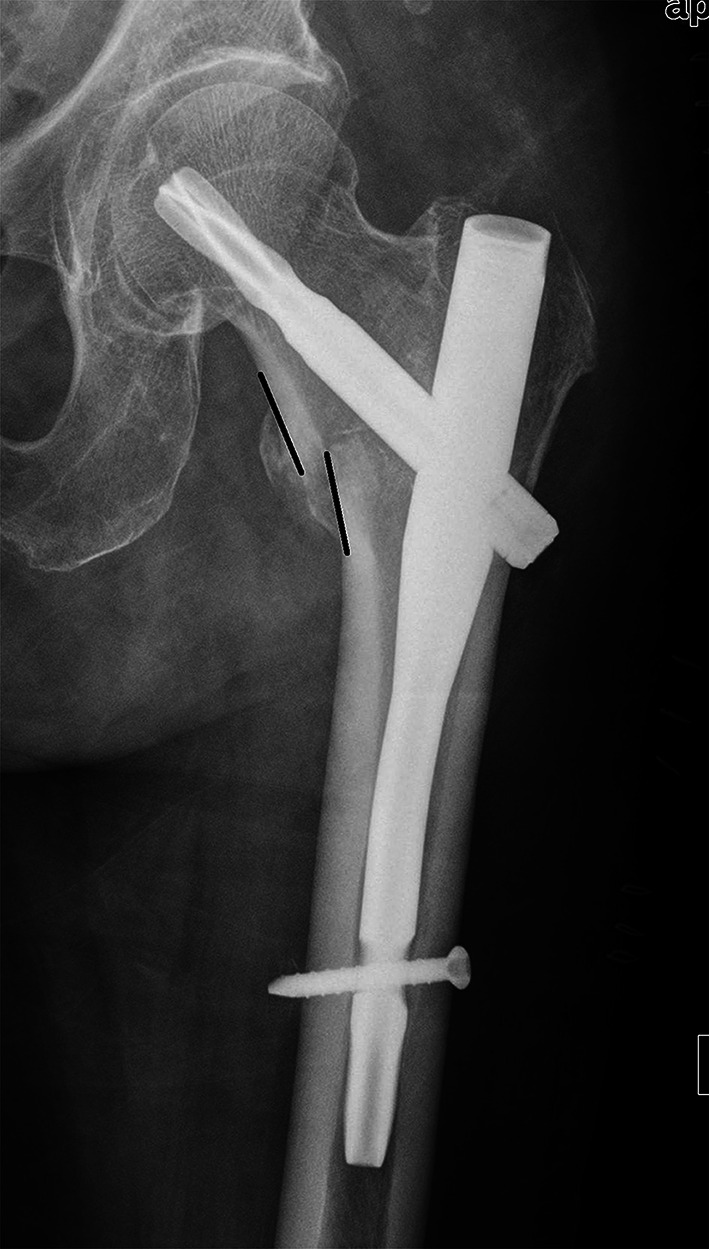
Positive medial cortex support (PMCS): the proximal femoral head–neck fragment is displaced medially to the upper medial edge of the distal femoral shaft fragment

**Fig. 2:**
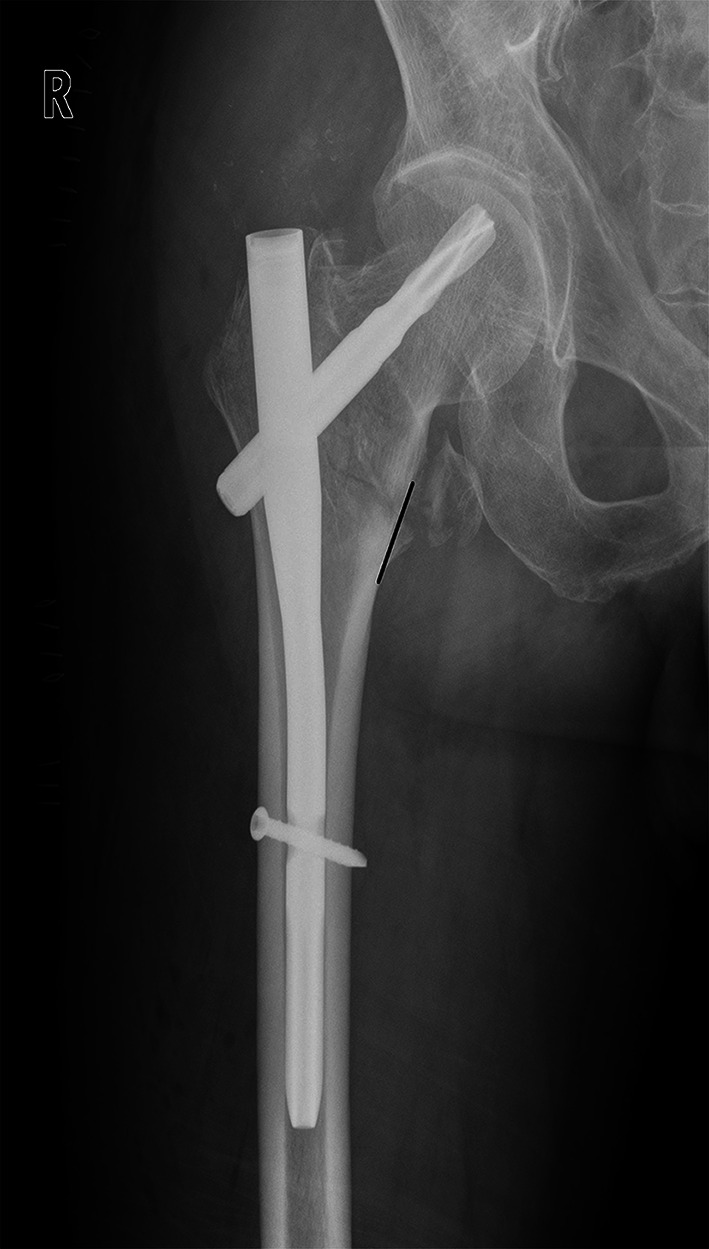
Neutral position (NP): the medial cortex of head–neck and the shaft fragments are smoothly contacted

**Fig. 3:**
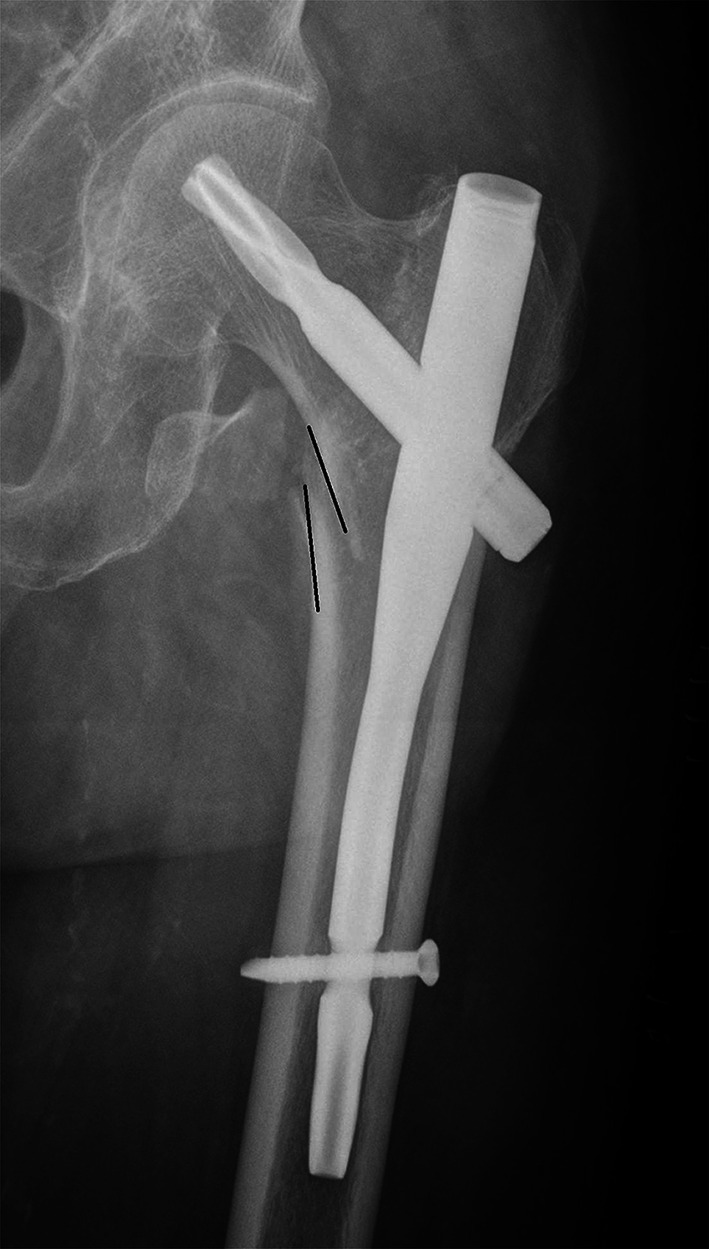
Negative medial cortex support (NMCS): the head–neck fragment is displaced laterally to the upper medial edge of the shaft fragment, which lost the medial cortex support from the femoral shaft

A full description of anteromedial reduction, or cortical support reduction, involved the assessment in both AP view (for medial cortex) and lateral view (for anterior cortex). In AP view, we use a new criterion to classify the quality of fracture reduction, via the position of the medial cortex between the femoral head–neck fragment and the shaft. (1) Positive medial cortex support (PMCS): the proximal femoral head–neck fragment is displaced medially to the upper medial edge of the distal femoral shaft fragment, i.e. the medial cortex of the head–neck fragment is located a little bit (one cortex thickness) superomedially to the medial cortex of the femoral shaft (Fig. 1). (2) Neutral position (NP): the medial cortex of head–neck and the shaft fragment are anatomically contacted (Fig. 2). (3) Negative medial cortex support (NMCS): the head–neck fragment is displaced laterally to the upper medial edge of the shaft fragment, which lost the medial cortex support from the femoral shaft (Fig. 3).

In lateral view, we assessed the relationship between the two anterior cortices of head–neck and shaft fragments into two categories. If the anterior cortices contacted smoothly or the step-off was less than 2 mm or half of the cortex thickness, it is classified as “Yes” anterior cortical support. If the head–neck cortex was posteriorly displaced more than 2 mm or half of the cortex thickness, it is classified as “No” anterior cortical support.

Radiographs were taken for evaluation of fracture union and implant related complications (cut-out, telescope and failure) at 3 and 6 months after surgery. In follow-up, we measured two parameters to determine fracture impaction. (1) The femoral neck–shaft angle, which is the angle between the two axes of head–neck and shaft medullary. (2) The length of the femoral neck, which is the distance between head center and shaft medullary center along the head–neck central axis.

### Data analysis

All statistical calculations were performed using SPSS version 19.0 (SPSS Inc. Chicago, IL, USA). Basic descriptive statistical analyses were used to describe the patient population and treatment outcomes. Student *t* test was used for continuous data and Fisher exact test or Pearson’s Chi-squared test for categorical variables. Statistical significance was defined as *p* < 0.05.

## Results

According the relationship of medial cortex position in AP radiographs, there were 89 cases (70 %) in positive support, 26 (20.5 %) in neutral support, and 12 (9.5 %) in negative support. There were no statistical differences among the three groups in age, sex ratio, prefracture score of activity of daily living (pre-ADL), walking ability score (WAC), ASA physical risk score, number of medical comorbidities, osteoporosis Singh index, and the position of lag screw or helical blade in femoral head (TAD). The demographics and operative data are given in Table 1.

**Table 1:** Patient demographics and operative data

	PMCS	NP	NMCS
Cases	89	26	12
Age	81.0 (68–97)	81.5 (69–93)	82.3 (73–92)
Male/female	22/67 (F: 75.3 %)	7/19 (F: 73.1 %)	12/8 (F: 66.7 %)
Prefracture BADL	15.1 (14–16)	15.3 (14–16)	15.1 (13–16)
Prefracture WAS	7.7 (5–9)	7.8 (5–9)	8.0 (5–9)
ASA grade 3/4	74/89 (83.1 %)	22/26 (84.6 %)	10/12 (83.3 %)
Medical comorbidities (>3)	45/89 (50.7 %)	15/26 (57.7 %)	7/12 (58.3 %)
Osteoporosis (Singh index 1–3)	67/89 (75.3 %)	19/26 (73.1 %)	9/12 (75.0 %)
Fracture type(AO/OTA)			
31A2.2	32	8	3
31A2.3	57	18	9
Baumgaetner fracture reduction criteria: poor	4/89 (4.5 %)	2/26 (7.7 %)	1/12 (8.3 %)
TAD >25 mm	5/89 (5.6 %)	2/26 (7.7 %)	1/12 (8.3 %)
Iatrogenic lateral wall broken	9/89 (10.1 %)	3/26 (11.5 %)	1/12 (8.3 %)
Secondary surgery due to implant failure	0	0	0

*PMCS* positive medial cortical support, *NP* neutral position, *NMCS* negative medial cortical support

At 3 months follow-up, there was minimal difference in radiograph measurement between the injured and the normal contralateral extremity, both in PMCS and in NP groups. As for the NMCS group, the mean neck–shaft angle and the femoral neck length lost significantly compared to the normal side.

The mean loss of the femoral neck–shaft angle in PMCS, NP and NMCS groups was 0.7°, 4.8°, and 8.9°, respectively. The differences among these three groups were statistical significant. The same trend was presented in the neck shortening, which was 2.4 mm in PMCS group, 3.5 mm in NP group and 6.7 mm in NMCS group. The PMCS group had least loss both in femoral neck–shaft angle and neck length. Compared with NMCS group, PMCS group also got ground-walking (full-weight bearing walking) much earlier, with better functional outcome at 3 months follow-up and less hip–thigh pain presence (Table 2).

**Table 2:** Postoperative follow-up data

	PMCS	NP	NMCS
Neck–shaft angle			
Postoperation*	135.2° (130–142)	135.7° (131–139)	131.3° (125–135)
3 months follow-up**	134.5° (128–142)	130.9° (125–137)	122.4° (117–125)
Contralateral limb	130.7° (127–133)	129.6° (126–132)	129.9° (127–132)
The length of femoral neck			
Postoperation*	46.8 mm (44–48)	45.6 mm (43–47)	42.5 mm (40–44)
3 months follow-up**	44.4 mm (43–47)	42.1 mm (41–46)	35.8 mm (33–40)
Contralateral limb	43.3 mm (41–46)	43.5 mm (40–47)	44.0 mm (41–47)
Timing of full-weight bearing (week)*	4.7 (4–6)	4.9 (4–7)	7.6 (6–10)
Postoperative BADL score			
3 months*	11.2 (9–11)	10.4 (7–11)	8.7 (7–10)
6 months	13.8 (11–16)	13.4 (9–15)	12.5 (8–15)
Postoperative WAC			
3 months**	6.9 (5–8)	6.2 (5–7)	5.2 (4–7)
6 months	7.7 (7–9)	7.5 (6–9)	7.1 (4–9)
Hip–thigh pain			
6 months	8 (9 %)	3 (11.5 %)	2 (16.7 %)

Comparison was made between PMCS and NMCS groups. * *p* < 0.05, ** *p* < 0.01

## Discussion

In the operation of unstable pertrochanteric fractures, anatomic reduction is always prior to the recommended positions of variety implants. Although the posteromedial cortex alignment is the key for successful reduction, most implants used today do not have the ability to purchase the less trochanteric fragment. According to the reduction criteria modified by Baumgaetner, most unstable fractures (31A2-3) could only be achieved “acceptable” reduction grade, i.e. good alignment. For these fractures, the Garden alignments and anteromedial contact between the femoral head–neck and shaft fragments are extremely important [1]. However, valgus position in fracture alignment is not synonymous to positive medial cortical support in fragment displacement.

Compression of the bone fragments is beneficial to bone healing. For unstable pertrochanteric fractures, it can be achieved through two approaches: intraoperative fracture compression and postoperative impaction via controlled sliding along the axis of the instrument device (helical blade or lag screw). The former is the maneuver done by the surgeon during surgery to compress the fracture site through which to obtain primary fracture stability, while the latter is the postsurgical compression provided by a fixation device with a sliding capability, in association with muscle contraction and patient weight bearing, attained secondary fracture stability.

Controlled fracture impaction by limited sliding, provides secondary axial and torsional stability between the head–neck fragment and the femur shaft. Controlled fracture impaction is particularly important for the maintenance of stable reduction during fracture healing, and is compatible with the subsequent dynamic events of cyclic loading and remodeling across the fracture line. In contrast, fracture collapse, also termed uncontrolled fracture impaction, or excessive sliding, is fracture impaction-displacement, with loss of reduction. Fracture collapse is one of the major reasons for failure of fixation of these fractures.

The concept of nonanatomic positive cortex buttress reduction was firstly introduced by Gotfried [20, 21] for displaced subcapital femoral neck fracture. On the premise of 180° fracture alignment in lateral view, it was defined a displaced subcapital femoral position, AP view, in which the distal femoral neck fragment is positioned medially to the lower-medial edge of the proximal fracture fragment. In this state, the distal fragment can limit the femoral head excessive sliding through cortex-to-cortex buttress [22].

**Fig. 4:**
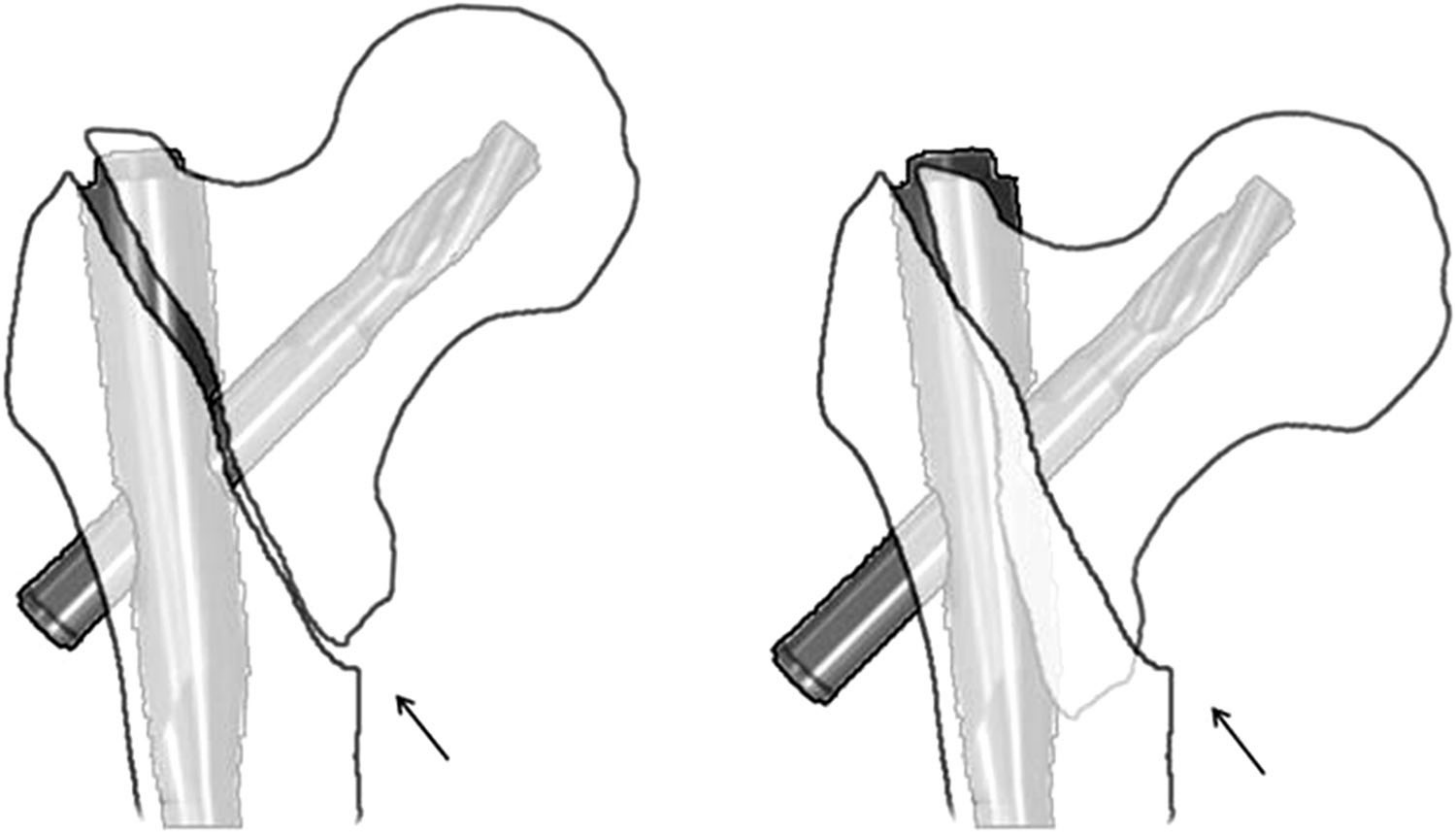
Schematic drawing for NMCS: proximal fragment impacted into the comminuted and low-density trochanteric region until touching the fixation nail. *Arrows* show the cortex–cancellous contact

**Fig. 5:**
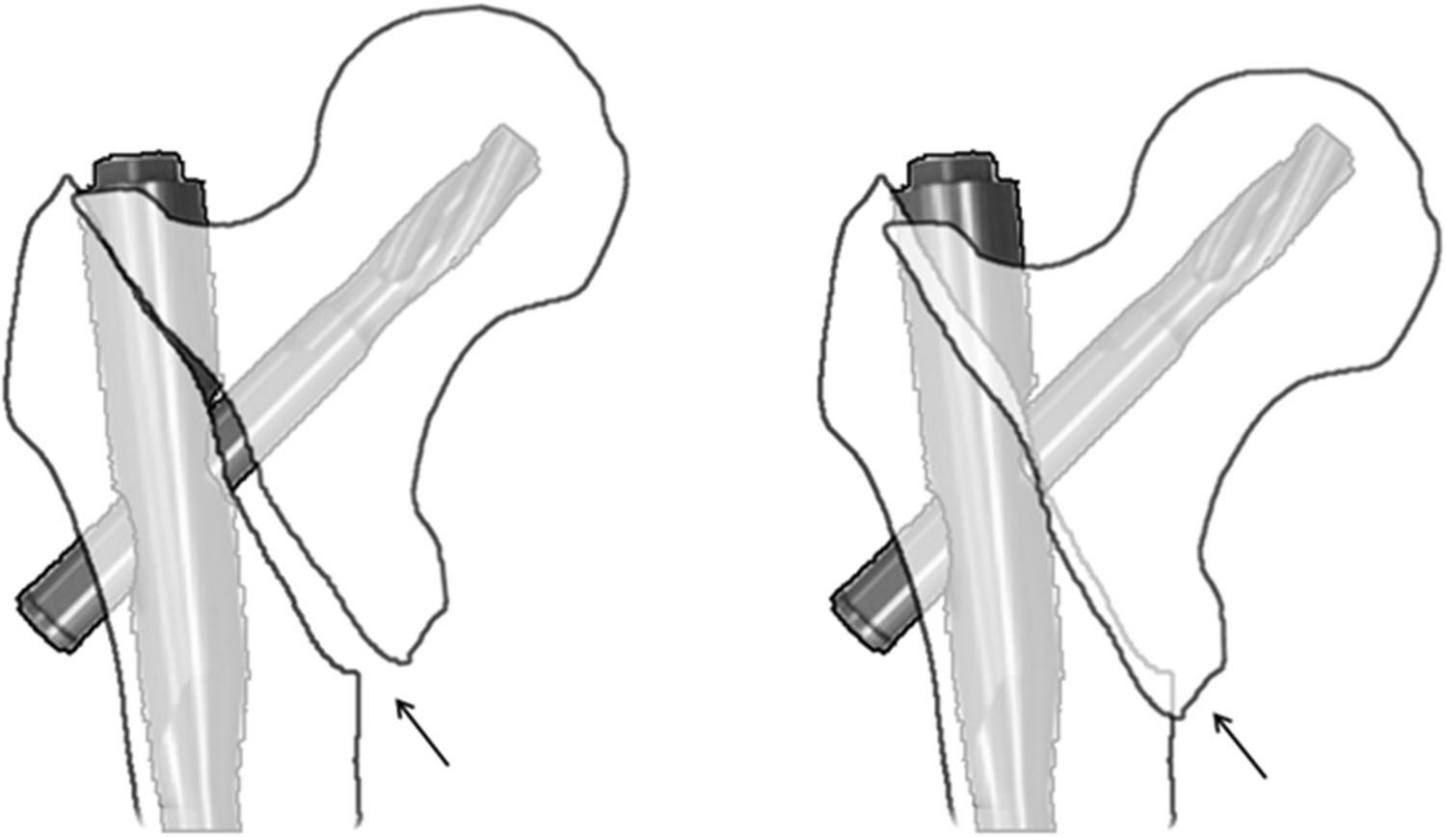
Schematic drawing for PMCS: the medial cortex of shaft resists proximal fragment from further sliding laterally. *Arrows* show the cortex–cortex contact

We present a counterpart concept of positive medial cortical support in unstable pertrochanteric fractures. It also demand a 180° fracture alignment in lateral view, while in AP view, contrary to the Gotfried’s standard, the distal femoral shaft fragment is intentionally positioned a little bit laterally to the lower-medial edge of the proximal fracture fragment. Unlike the usual displaced route of the proximal fragment in unstable femoral neck fractures, for pertrochanteric fractures, when sliding begins after surgery, the head–neck fragment is tended to displace laterally, impacted into the comminuted and low-intensity trochanteric region, which finally led to collapse (Fig. 4). As in positive medial cortical support position, the cortex contact between the two main fragments are achieved, meanwhile, the medial cortex of the femoral shaft can resist the femoral head–neck fragment from further sliding laterally (Fig. 5). The anterior cortical contact after head–neck sliding can also provide rigid buttress for secondary stability [23, 24]. However, considering the essence of lateral sliding direction, we think positive medial cortical support maybe more effective than anterior cortical contact [14]. In addition, obtaining both medial and anterior cortical buttress (anteromedial reduction) is the best option for pertrochanteric fragment reduction.

However, exact anatomic reduction of anteromedial cortex is rare in reality. The so-called “anatomic reduction” shown in intraoperative fluoroscopy may actually contain three sub-conditions: some are in exact anatomic cortex-to-cortex position, others in slight positive position, and still others in slight negative position. But as the image resolution was limited, those sub-conditions were hardly to be distinguished clearly. So we used the term “neutral” to instead “anatomic”. After bone resorption of the fracture line, slight negative position might become truly negative position. In our case series, five patients with neutral cortical reduction (5/26, 19 %) became negative reduction later, and their outcomes were lower. In lateral radiographs, these five cases also lost their anterior cortical support. The neck cortex was located posteriorly to the shaft cortex more than one cortical thickness. The other 21 cases had a real anatomic reduction of the medial cortex, and obtained a PMCS.

In our series, using cephalomedullary nail seems easy to get a positive medial cortical support reduction (89/127, 70 %). One possible explanation is that pertrochanteric fracture is a kind of extracapsular fracture, the traction applied to the leg can relatively easy to separate the two main fragments. When insert the nail from the medial edge of the greater trochanter, wedge-open effect [25] may occur between the femoral head–neck fragment and the lateral wall, the nail may push the lateral wall, and move the shaft laterally, which makes the shaft fragment be positioned laterally to the lower-medial edge of the proximal head–neck fragment. Positive medial cortex support reduction and wedge-open effect can increase the femoral off-set theoretically, which is beneficial to the strength of the abductor muscles. However, over distraction and/or open (greater than one cortex) may decrease the impaction area among the fragments, lead to delayed union or nonunion.

**Fig. 6:**
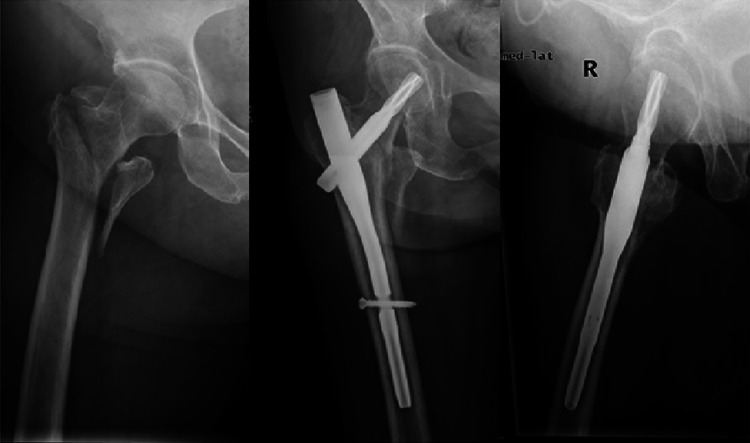
Excellent quality of fracture reduction. Slight valgus and 180° for alignment, positive cortical support and smooth anterior cortical contact for displacement in AP and lateral radiography were achieved

Now in practice, for unstable pertrochanteric fractures, we attempt to achieve an ideal anteromedial reduction between the head–neck and shaft fragments, i.e. slight valgus and 160°–180° for alignment, positive or anatomic medial cortical support and smooth anterior cortical contact for displacement, in AP and lateral radiography, respectively (Fig. 6).

Our recommendations for good quality of fracture reduction (Table 3), include slight valgus position in alignment and positive medial cortical support in displacement (AP view), and central axial alignment with smooth anterior cortex contact (sagittal view).

**Table 3:** Quality of fracture reduction between head–neck fragment and femoral shaft

Items	Scores
Garden alignment	
AP view: slight valgus or normal	1
Lat view: 160°–180°	1
Fragment displacement	
AP view: positive or neutral medial cortex support	1
Lat view: anterior cortex smooth continuity	1
Quality of fracture reduction	
Excellent	4
Acceptable	3 or 2
Poor	1 or 0

In conclusion, fracture reduction with positive medial cortical support and valgus alignment, allows limited sliding of the head–neck fragment to contact with the femur shaft and achieve secondary stability, providing a good mechanical environment for fracture healing.
